# To Treat or Not to Treat: A Scoping Review of Speech Treatment for Dysarthria in Amyotrophic Lateral Sclerosis (ALS)

**DOI:** 10.3390/healthcare13192434

**Published:** 2025-09-25

**Authors:** Brooke-Mai Whelan, Danielle Aldridge, Jessica Ruhle, Persephone Whitelock, Shana Taubert, Annette Collins, Elaine Kearney, Salma Charania, Robert D. Henderson, Sarah J. Wallace, Claire Mitchell, Kaila L. Stipancic, Mili Kuruvilla-Dugdale, Adam P. Vogel

**Affiliations:** 1 School of Health and Rehabilitation Sciences, Faculty of Health, Medicine and Behavioural Sciences, The University of Queensland, Brisbane, QLD 4072, Australia; d.aldridge@uq.edu.au (D.A.); p.whitelock@student.uq.edu.au (P.W.); elainekatrina.kearney@uq.edu.au (E.K.); s.wallace3@uq.edu.au (S.J.W.); 2 Royal Brisbane and Women’s Hospital, Brisbane, QLD 4029, Australia; shana.taubert@health.qld.gov.au (S.T.); annette.collins@health.qld.gov.au (A.C.); robert.henderson@health.qld.gov.au (R.D.H.); 3 The Princess Alexandra Hospital, Brisbane, QLD 4102, Australia; 4 School of Health, Medical and Applied Sciences, Central Queensland University, Rockhampton, QLD 4701, Australia; s.charania@cqu.edu.au; 5 University of Queensland Centre for Clinical Research, The University of Queensland, Brisbane, QLD 4029, Australia; 6 Psychology, Communication and Human Neuroscience, School of Health Sciences, Geoffrey Jefferson Brain Research Centre, University of Manchester, Manchester M13 9PL, UK; claire.mitchell@manchester.ac.uk; 7 Department of Communicative Disorders and Sciences, University at Buffalo, Buffalo, NY 14214, USA; klstip@buffalo.edu; 8 Department of Communication Sciences and Disorders, The University of Iowa, Iowa City, IA 52242, USA; mkuruvilladugdale@uiowa.edu; 9 School of Health Sciences, The University of Melbourne, Melbourne, VIC 3010, Australia; vogela@unimelb.edu.au; 10 Redenlab, 585 Little Collins Street, Melbourne, VIC 3000, Australia

**Keywords:** dysarthria, treatment, motor neurone disease, speech, amyotrophic lateral sclerosis

## Abstract

Background: Speech loss is recognised as one of the most devastating outcomes for individuals with ALS, yet active speech intervention is rarely targeted in this population. Clinicians face significant challenges in managing dysarthria associated with ALS due to the rapidly progressive nature of the disease, historical concerns around intensive exercise accelerating decline, and an absence of direction on restorative and compensatory intervention strategies in current clinical care guidelines. This review evaluates the scope and quality of evidence for speech treatments in ALS to identify knowledge gaps and establish research priorities to guide clinical care. Methods: Studies were retrieved from six electronic databases (PubMed, CINAHL, Embase, Cochrane library, Web of Science, and PsycINFO). Results: Four studies met inclusion criteria. Treatment approaches included: music-based speech therapy; multisubsystem speech rehabilitation program, tongue strengthening and articulation training; and Lee Silverman Voice Treatment-LOUD^®^ combined with additional voice and articulation therapy. Sample sizes were small, with all studies demonstrating notable methodological weaknesses. The limited evidence base, marked by conflicting results and methodological flaws, prevents any reliable conclusions about treatment effectiveness. Conclusions: Despite the prevalence and impact of dysarthria in this population, evidence for speech treatment remains sparse, of generally low quality, and provides limited guidance for clinical practice. The changing perspective on exercise in ALS warrants rigorous investigation of tailored dysarthria interventions for this population that are minimally fatiguing and enhance speech by making use of residual physiologic support.

## 1. Introduction

The rapid progression of dysarthria in amyotrophic lateral sclerosis (ALS) is largely unrivalled by any other neurodegenerative disease. Speech changes frequently occur prior to a diagnosis [1] and the ability to speak may be lost within 18 months of initial symptom presentation [2]. Bulbar-onset ALS is recognized as the disease variant with primary implications for speech functioning [3], with dysarthria reported in up to 93% of individuals in this group [4]. Although the bulbar-onset variant is commonly associated with dysarthria at symptom onset, it is estimated that 80% of individuals with the spinal variant will develop bulbar symptoms over the course of disease progression [5]. These findings underscore that deterioration of speech function is commonly observed in ALS across disease phenotypes.

ALS involves the progressive loss of both upper and lower motor neurons [6] leading to mixed spastic-flaccid dysarthria. Mixed dysarthria in ALS is characterised by consonant imprecision, hypernasality, vocal harshness and reduced speech rate [7]. This profile is the result of impairments across multiple speech subsystems, yet the implication of specific subsystems and associated rates of deterioration vary across individuals and disease stage [3,8,9]. Predictive models of speech loss in ALS propose a hierarchical pattern of subsystem involvement, beginning with changes in articulation and phonation (often prior to alterations in intelligibility or rate), followed by subsequent involvement of the velopharyngeal and respiratory subsystems as the disease progresses [10]. Findings from early behavioural studies of speech decline in ALS, however, reported early vulnerability of the velopharyngeal and phonatory subsystems in ALS [9], suggesting that the pattern of dysarthria progression is not uniform across individuals. Notably, people with ALS have identified dysarthria progression and associated speech loss as the most distressing aspect of the disease, superseding the inability to walk, swallowing difficulties, and the awareness of a terminal prognosis [11]. A clinical imperative, therefore, is to provide timely and effective speech interventions to preserve natural speech in people with ALS for as long as possible.

Current clinical care guidelines for the management of communication in ALS advocate for compensatory speech strategies and augmentative and alternative communication (AAC) systems, excluding restorative speech intervention protocols for dysarthria [12,13,14]. Within this context, clinicians largely avoid the use of restorative intervention protocols that involve intensive manipulation of speech subsystems through specific tasks and repeated exercises designed to modify underlying motor patterns of speech production [15]. The exclusion of restorative speech interventions in care guidelines perhaps reflects the rapidly progressive nature of ALS, necessitating a management shift from speech-oriented to communication-oriented approaches, which involves pivoting from the active restoration of natural speech over time, to maintaining communication via methods with immediate functional effect. Alternatively, the lack of specific guidance around restorative speech treatments may reflect historical perspectives that caution against exercise-based treatments in ALS, due to their perceived potential role in inducing fatigue, accelerating disease progression and subsequently, functional decline [16,17]. Early publications within the field of speech pathology cautioned against therapeutic speech exercise programs in ALS [18], citing evidence of dysarthria progression following intervention attempts in early case studies [19,20].

Recent reviews, however, suggest that tailored therapeutic exercise may be beneficial in ALS, challenging previous assumptions. For example, limb and respiratory exercises delivered at mild to moderate intensities in early-stage ALS, have the capacity to maintain or improve motor and pulmonary functioning [21,22]. These emerging insights about therapeutic exercise in ALS have potential implications for the management of dysarthria in this population. In other neurodegenerative conditions, specifically Parkinson’s disease, a tailored speech exercise protocol targeting respiratory-laryngeal subsystems has been rigorously investigated and shown to unequivocally improve vocal loudness [23,24,25], with some preliminary evidence of distributed effects to articulation [26,27], facial expression [28,29], and swallowing [30,31]. Similarly, improved speech intelligibility and naturalness following a tailored speech treatment program for individuals with multisystemic degenerative ataxia, suggests that structured speech exercise may also be effective in managing dysarthria associated with degenerative cerebellar disease [32,33]. Despite acknowledged differences in the underlying pathophysiology and rate of disease progression in ALS, the positive treatment outcomes observed in other neurodegenerative dysarthrias provide an empirical foundation for exploring structured speech exercise protocols in ALS.

Considering the changing viewpoints on exercise-based therapies in ALS, and evidence of positive speech treatment outcomes in other neurodegenerative populations, this review aimed to (1) synthesise current evidence for dysarthria intervention in ALS based on studies reporting pre- and post-dysarthria treatment data; (2) identify knowledge gaps; and (3) set future research directions to inform clinical practice.

## 2. Methods

A systematic scoping review was undertaken to identify and analyse the available evidence pertaining to speech treatments for dysarthria in ALS, with a focus on speech training protocols. Scoping review methodology was employed to characterize the existing body of research relating to dysarthria treatment for ALS, guided by the recommendations of the Joanna Briggs Institute (JBI) for the conduct of systematic scoping reviews [34]. This review is reported against the Preferred Reporting Items for Systematic Reviews and Meta-Analyses Extension for Scoping Reviews (PRISMA-ScR) (see Supplementary File S1) [35]. There is no published protocol for this review.

### 2.1. Participants

The population of interest included adults of any gender aged 18 years or older with a confirmed diagnosis of ALS and dysarthria.

### 2.2. Concept and Context

This review focused on speech treatments for dysarthria in ALS, specifically examining interventions involving structured speech training/exercise protocols. Structured speech training protocols were defined as those comprised of exercise that was planned and repetitive, with the aim of improving physical performance (i.e., speech production) over time [36]. There was no restriction on the type or severity of dysarthria, duration of speech treatment, disease duration, or medication regimen. Additionally, there were no restrictions on the location of participants (e.g., home, research facility, hospital) during speech treatment. The reporting of baseline and post-treatment speech outcome measures was required and were permitted to include measures of impairment, activity, or participation, or a combination of measures relating to these classifications of functioning. All study designs (e.g., quantitative, qualitative, controlled trials) were considered.

### 2.3. Search Strategy and Study Selection Criteria

Initially, a small selection of relevant articles accessed via PubMed were used to identify key words and index terms to form the basis for development of a comprehensive search strategy. Search terms were generated under three central topic areas including ALS, speech impairment/dysarthria, and speech rehabilitation/exercise. An independent librarian DH) based at The University of Queensland, Australia provided peer review of the search strategy design and search strings. Utilizing the Polyglot Search Translator [37] within the Systematic Review Accelerator software [38], final search strings were generated (see Supplementary File S2) and applied to six databases including PubMed, CINAHL, Embase, Cochrane library, Web of Science, and PsycINFO.

Selection criteria were pre-defined to include any study investigating speech treatments (as defined above) for people with ALS that was available in English and uploaded to one of the six databases prior to mid-May 2025. Conference abstracts were excluded on the basis of limited methodological detail and insufficient data reporting to provide meaningful quality assessment and synthesis of findings. Similarly, opinion papers, letters to the editor/editorials and book chapters with no clear methods or results were excluded, as were dissertations, theses, and other review articles. Studies in which fewer than 50% of participants had a diagnosis of ALS were required to present disaggregated data, allowing for separate examination of outcomes specific to participants with ALS.

Records returned from the database searches were imported into EndNote where duplicates were removed before the remaining records were uploaded into Covidence © systematic review management software [39]. Any remaining duplicates were automatically purged by the software before screening of titles and abstracts took place. Under the supervision of a qualified speech pathologist and experienced researcher (BMW), three research assistants (DA, JR & PW) independently screened all available titles and abstracts available until mid-May 2025, to identify any records for full text review that showed conformity with the pre-determined selection criteria. Any disagreements between the reviewers were resolved through discussion with BMW referencing the pre-established selection criteria. Following initial screening, full texts of studies meeting preliminary criteria were retrieved and uploaded into Covidence ©. Independent evaluation of each full text study was conducted by DA and BMW against the selection criteria to determine final inclusion. Where there was uncertainty or disagreement, consensus was reached through discussion between DA and BMW. In a final step to ensure all relevant studies had been identified, hand-searches were carried out on the reference lists from included studies. No additional records were identified through this process.

### 2.4. Data Extraction, Analysis, and Reporting

Data extracted from the identified studies investigating speech treatment associated with dysarthria in ALS included: primary author(s); year of publication; country of origin; study aims; participant characteristics; sample size; study design; speech treatment description; speech outcome measures; key findings; level of research evidence. Both DA and BMW were involved in extracting and analyzing the data from the final selection of studies. The level of the research evidence was determined according to the National Health and Medical Research Council of Australia (NHMRC) evidence hierarchy [40] and interpreted according to the grades of recommendations for the development of evidence-based clinical guidelines defined by the NHMRC [41]. Methodological quality was also systematically assessed for all studies by two reviewers (DA, BMW) using a structured evaluation approach (i.e., JBI critical appraisal checklists for case series [42] and case reports [43]). The JBI case series checklist (for studies sampling participants with a specific disease or disease-related outcome) evaluates ten study criteria including: clarity of inclusion criteria, standardized measurement methods, method for identifying clinical condition, consecutive participant inclusion, complete recruitment, demographic reporting (participant and site), clinical information, outcome reporting, and the appropriateness of statistical analysis. The JBI case report checklist (for single participant studies) assesses eight criteria focusing on demographic description, patient history timeline, clarity of clinical condition, diagnostic testing, intervention procedures, post-intervention outcomes, adverse event reporting, and provision of meaningful takeaway lessons.

## 3. Results

### 3.1. Screening and Identification

**Figure 1:**
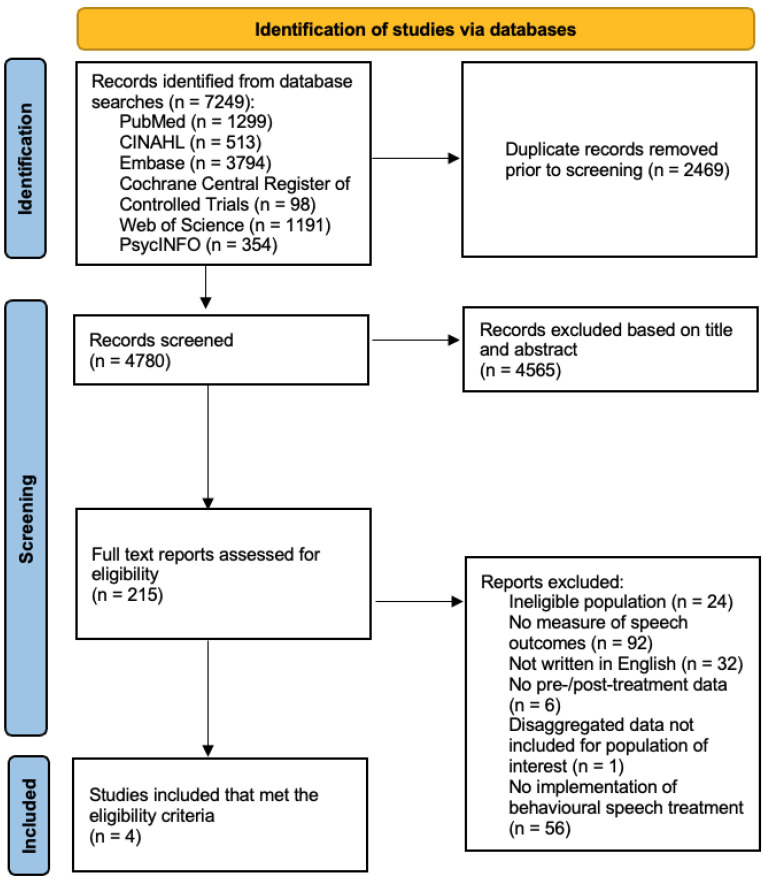
Flow diagram for study selection (adapted from Preferred Reporting Items for Systematic Reviews and Meta-Analyses: The PRISMA Statement [44]).

A total of 7249 papers were identified via database searches of which 2469 were duplicates (see Figure 1 for the preferred Reporting Items for Systematic Reviews and Meta-Analyses (the PRISMA statement)) [44]. Following removal of duplicates, 4780 records were screened based on title and abstract regarding their eligibility for inclusion in the review. A total of 4565 records did not match the pre-set selection criteria and were excluded for reasons not limited to but including: non-ALS study population; topics outside dysarthria intervention research (e.g., drug trials), non-English studies etc. The full text version of the remaining 215 records were evaluated against the selection criteria. Only 4 studies met the eligibility criteria for inclusion in this review. Two hundred and eleven studies were excluded based on: ineligible population (24), no reported measure(s) of speech treatment outcome(s) (92), not available in English (32), no pre-/post-treatment data (6), no disaggregated data available for population of interest (1) and, no implementation of speech treatment (56).

### 3.2. Types of Studies, Levels of Evidence and Context

Over the past 46 years (1979–2025), four speech treatments (summarised in Table 1) have been investigated in individuals with dysarthria associated with ALS including: (1) music-based speech therapy (integrating postural, breath support, resonance, intonation, articulation, and vocal projection exercises) [45]; (2) an integrated multisubsystem speech rehabilitation program targeting respiration, resonance, prosody, and phonation [46]; (3) tongue strengthening exercises and articulation training [19]; and (4) the Lee Silverman Voice Treatment (LSVT)-LOUD^®^ program in combination with resonant voice, isometric vocal fold exercise, and articulation therapy [20]. Of the four studies identified, one was an interrupted time series without parallel controls (Level III-3), and three were single case series designs (Level IV) [40] (see Table 1). The methodological quality of the four included studies varied considerably based on JBI critical appraisal criteria [43], ranging from high (88%) to low (38%) (see Table 1), indicating substantial variability in study rigor.

Across studies, sample sizes were small ranging from one to seven participants with ALS. All studies followed a repeated measures design and incorporated pre- and post-treatment speech/communication outcome data. Included studies were conducted in Russia, Brazil, and the United States of America.

### 3.3. Speech Treatments, Aims, and Outcomes

#### 3.3.1. Music-Based Speech Therapy

Apreleva [45] and colleagues administered in-home, individualized music-based speech therapy (MT) for one hour, twice weekly, over six weeks to seven participants with ALS. The study aimed to evaluate the feasibility and efficacy of the MT treatment program for people with ALS. The therapy focused on increasing breath support, promoting muscle relaxation and faster speech rate, decreasing hypernasality, and maintaining swallowing co-ordination. Participants were provided with written educational information aboutvocal health and ALS-specific healthy voice practices for daily life. The MT treatment protocol included an opening assessment, body alignment exercise, diaphragmatic breathing exercises, controlled breathing and lip seal exercises, music-assisted relaxation for voice production, soft palate exercises, phonation exercises, consonant range cantillation exercises, velopharyngeal port exercises, impulse diaphragmatic breathing exercises, sustained vowel production exercises, laryngeal elevation through vocalization (gliding vowels) exercises, vocal cord relaxation exercises, song performance, and final assessment before session closure. Participants were encouraged, but not required, to practice the skills learned in their MT sessions on non-intervention days.

Speech outcomes were recorded at four time points: baseline (Week 1), pre-treatment (Week 6), post-treatment (Week 12), and follow-up (Week 16). Despite reported trends of sustained or improved function across a range of bulbar and respiratory measures, no statistical analyses were performed on this data. As such, these results warrant cautious interpretation and will not be explored further in this review. Feasibility was an important aspect of this study, with both quantitative data, and qualitative themes from semi-structured interviews carried out with participants and carers. Preliminary evidence indicated that a home-based speech treatment program was acceptable to people with ALS supported by high retention and treatment adherence rates.

The Apreleva et al. [45] study met seven of the ten JBI critical appraisal criteria for case series. Evident strengths of this study included clear inclusion criteria consisting of a confirmed diagnosis of probable or definite ALS by a neurologist; an ALS Functional Rating Scale-Revised (ALSFRS-R) bulbar score of greater than or equal to 9 but less than or equal to 11 [47]; forced vital capacity of greater than 60%; unimpaired cognition (determined by screening with Edinburgh Cognitive and Behavioural ALS Screen) [48]; ability to consent to treatment; and Russian as native language. A valid method for identifying the condition for all included participants was provided (i.e., revised El Escorial criteria confirmed by neurologist) [49]. The case series utilized a consecutive sampling strategy and reported participant demographic data (e.g., gender and age) and recruitment site (e.g., ALS Centre Moscow (Russia)). Failure to clearly report comprehensive clinical information for all participants was an identified limitation of this study. For example, no information regarding disease duration, dysarthria severity, or motor speech subsystem impairments was provided. Furthermore, there was an arbitrary cessation of recruitment after achieving a sample size of eight, despite all newly diagnosed patients with ALS in the metropolitan area fitting inclusion criteria being invited to participate. There was no clear justification for this sample size and descriptive statistics were used exclusively to describe trends in the dataset. While the study utilized standardized assessment protocols for feasibility (e.g., adherence (number of sessions attended)), tolerance (e.g., self-reported ease of respiration rating scale), home-practice monitoring) and biomedical (e.g., forced vital capacity, maximal inspiratory and expiratory pressure, speech acoustic assessment) outcomes across participants, the high number of outcome variables tested relative to the small sample size represented a significant methodological flaw.

#### 3.3.2. Integrated Multisubsystem Rehabilitation Program

Rohers et al. [46] implemented an integrated multisubsystem speech rehabilitation program for people with neurodegenerative conditions and employed a communication-related quality of life scale and frequency-following response (FFR) measures to evaluate therapeutic response. The FFR response is an auditory evoked electrophysiological potential associated with central auditory processing [46]. Electrophysiological measures such as the FFR have been reported to offer a means of quantifying neuroplastic adaptations in auditory processing [50]. Within the context of dysarthria treatment, alterations in FFR response following intervention may be indicative of changes in the neural coding of speech [51], concurrent with changes in intelligibility [52]. Initially, recruitment involved 11 people with neurodegenerative conditions and dysarthria, nine of whom were subsequently excluded due to SARS-CoV-2 outbreak. Two male participants completed the study, one with a diagnosis of ALS and one with Parkinson’s disease. The speech treatment protocol included 25 sessions of 45 min each, over a six-month period. Five sessions were dedicated to one of four targeted individual speech subsystems (i.e., respiration, resonance, prosody, phonation) with an additional five sessions allocated to joint intervention and rehabilitation. Pre- and post-treatment implementation, participants completed a self-assessment (Living with Dysarthria [53]) questionnaire and underwent electrophysiological assessment using the FFR. Evaluations were carried out by separate assessors blinded to the participants’ treatment stage (i.e., pre- or post-treatment). Descriptive statistics indicated reductions in perceived speech-related difficulties on the self-assessment questionnaire total scores obtained for both participants following dysarthria therapy. Reduced scores (indicative of improved function) were observed across all domains explored by the questionnaire (e.g., speech problems, language/cognition problems, tiredness, effects on emotions, effects on different people, effects on different situations). In relation to FFR responses, the participant with ALS showed less benefit from the dysarthria intervention than the participant with Parkinson’s disease. However, increases in the amplitude of certain FFR waves seen for both participants were interpreted to have a potential association with increasing synapses and neuronal activations which may have resulted from the speech treatment.

This study met three of the eight JBI critical appraisal criteria for case reports, including clear reporting of demographic information (i.e., age and gender), assessment methods and results (i.e., pre- and post-treatment Living with Dysarthria questionnaire total score and FFR wave latencies and amplitude), and the reporting of the clinical implication that FFR shows potential as an objective measure for monitoring speech therapy progress in patients with dysarthria associated with neurodegenerative disease. Several limitations were identified in this study, including failure to provide a clearly delineated patient history with timeline or description of clinical condition (e.g., no information was provided in relation to disease duration/stage, dysarthria severity, onset type). In addition, limited information was provided around the speech treatment administered other than intensity of sessions and session focus (i.e., subsystem targeted), with ambiguity surrounding the joint rehabilitation and intervention component (i.e., lack of clarity around what subsystems were targeted in combination). Furthermore, description of the post-treatment clinical condition was limited by an absence of information relating to ALS progression or dysarthria severity, and the presence or absence of adverse events associated with treatment were not explicitly reported.

#### 3.3.3. Lee Silverman Voice Treatment (LSVT)-LOUD^®^, Vocal Deconstriction, Isometric Vocal Fold Exercises, and Articulation Therapy

Watts and Vanryckeghem [20] presented a descriptive case report of voice and articulation therapy provided to a 72-year-old female with ALS over a period of approximately four and a half months. Initially, the Lee Silverman Voice Treatment (LSVT-LOUD^®^) [54] was selected to address dysphonia. This treatment targeted low volume attributed to significant vocal fold bowing (confirmed by laryngoscopy) associated with an initial neurological diagnosis of multisystem atrophy (and later ALS), despite presence of moderate to severe compression of the ventricular folds and ventricular phonation when voicing. Initial LSVT-LOUD^®^ attempts resulted in deterioration of vocal quality and evidence of voice breaks associated with ventricular compression. A transition to forward resonance voice therapy was made to reduce laryngeal constriction. Successful reduction in laryngeal constriction was accompanied by an increase in breathiness and low volume. LSVT-LOUD^®^ was subsequently reintroduced but discontinued within 2 weeks because of severe ventricular phonation on attempts to increase loudness. Consequently, weekly treatment sessions of 1 and 1/2 h in duration were then initiated focusing on forward resonance, glottal fry techniques, and isometric vocal exercises (e.g., push-pull) to improve vocal quality. Forward resonance [55] and glottal fry [56] techniques were reported to reduce ventricular compression less than 50% of the time, and isometric exercises encouraging increased vocal loudness resulted in increased ventricular compression and reduced voice quality. No improvement was noted regarding voice quality following intervention, so the focus of therapy was shifted to articulation therapy involving daily oral motor mobility exercises and strengthening activities. Acoustic data revealed an increase in pitch and variable patterns in amplitude and frequency perturbation as well as noise to harmonic ratio metrics over the course of treatment. The reliability of these variable acoustic findings was questioned due to potential aperiodicity in the voice signal, limiting confidence in treatment effect interpretation. Sentence level intelligibility measures demonstrated a rapid decline in function across the 4 and ½ months therapy was delivered (i.e., 96% intelligible at baseline, declining to 10% intelligible at treatment termination). Quality evaluation of this study revealed compliance with seven of the eight domains of the JBI critical appraisal standards for case report studies. Despite clearly describing patient demographics, chronological history of presentation, pre- and post- intervention clinical condition, and adverse events, the authors failed to adequately describe the adjunct voice and articulation-based treatments provided to this patient. Furthermore, performance was not evaluated during the transition between therapies, limiting interpretation of outcomes. The take-home message from this research emphasized that the rate of disease progression in ALS may prohibit the ability to evaluate the efficacy of behavioral treatments in this population. This perspective overlooks an important methodological consideration, however, that robust natural history data may serve to establish critical baseline trajectories against which intervention effects can be differentiated from disease progression.

#### 3.3.4. Tongue Strengthening and Articulation Training

Dworkin and Hartman [19] described the effects of progressive lingual and velopharyngeal involvement on speech and swallowing in a 49-year-old male with ALS. The report described the introduction of tongue strengthening exercises and articulation training aimed at improving speech and swallowing function. The treatment protocol was not described in sufficient detail for replication. Although baseline and 6-month follow-up measures of tongue strength, mobility, and perceptual speech profiles were reported, no data immediately post-treatment was collected. Speech treatment was discontinued after one month due to extreme fatigue and breathing difficulties. While speech-related outcome measures were reported pre-treatment, the rapid nature of disease progression in this case precluded an evaluation of treatment efficacy. Six-month follow-up data (i.e.,4 months after termination of speech treatment) indicated significant deterioration in tongue strength and speed, in addition to moderate to severely reduced intelligibility and dysphagia. When evaluated against the comprehensive JBI quality assessment tool for case reports, this study met six of the eight criteria. This study described patient characteristics including clinical history, pre- and post-intervention clinical presentation, assessment methods and adverse events, however, failed to describe in detail what the applied tongue strengthening program (comprised of resistance exercises) and articulation training entailed.

## 4. Discussion

This scoping review identified a very limited body of evidence investigating behavioural speech treatment for dysarthria in ALS. Only four studies met the inclusion criteria, and none provided clear evidence of efficacy. This highlights the critical gap in evidence for speech therapy in ALS, despite the impact and widespread occurrence of dysarthria in this population. Two studies were conducted between 1979 and 2001 and only two within the last 24 years, highlighting very limited progress in this field across nearly five decades [19,20,45,46]. Treatment approaches varied across studies, and methodological quality was generally low, with no adequately powered trials.

Two of the four studies reported improvements in aspects of communication following speech treatment [45,46], and two studies documented declines in speech function over time, despite remediation attempts [19,20]. Of note, the two contemporary studies reported positive outcomes following speech treatment, contrasting with unfavourable results documented in earlier foundational research. Interestingly, earlier studies reporting less favourable outcomes demonstrated higher methodological quality. Caution is therefore warranted when interpreting these findings, as differences in research design and methodological quality may have contributed to the favourable outcomes reported in contemporary studies. The findings of this review are discussed through three critical perspectives relating to treatment implications: (1) deficit-specific versus integrated subsystems treatment approaches; (2) considerations for establishing appropriate therapeutic target hierarchies; and (3) the heterogeneity of bulbar deterioration in treatment selection.

### 4.1. Deficit-Specific Versus Integrated Subsystems Approaches

All four studies incorporated a motor speech subsystems (i.e., functional components) approach to treatment [57], however, the specific subsystems that were targeted varied across interventions. The functional components framework posits that motor speech control is orchestrated via five interdependent subsystems. Optimal treatment planning necessitates the accurate identification of speech features associated with specific subsystem deficits [15]. Intervention approaches typically target the most impaired subsystem/s first, recognising that changes at this level are more likely to have the greatest impact on speech function [15].

Two studies employed deficit-specific treatment approaches, focusing intervention exclusively on subsystems that demonstrated primary impairment at baseline evaluation [19,20]. For example, Dworkin and Hartman [19] tailored intervention to address lingual and velopharyngeal impairments identified during baseline assessment. Similarly, Watts and Vanryckeghem [20] initially implemented LSVT-LOUD^®^ after identifying respiratory and phonatory impairments as primary features of the presenting dysarthria as baseline, then sequentially introduced vocal deconstriction, isometric vocal exercises, and articulation therapy, to target remaining deficits.

Each of the above treatment approaches, however, failed to produce positive outcomes on speech function. In relation to the Dworkin and Hartman [19] study, treatment was discontinued within one month as a consequence of extreme fatigue and respiratory difficulty. As such, re-evaluation using impairment and activity-based measures was not possible until 4 months after cessation of treatment, where declines from baseline function were observed. According to the World Health Organisation’s International Classification of Functioning, Disability, and Health (ICF) framework [58], impairment-based speech assessments identify and describe underlying physiological or structural substrates impacting speech production (e.g., tongue strength) and activity-based assessments refer to how impairments of speech production affect an individual’s ability to perform speaking activities in daily life (e.g., holding a conversation). In the Dworkin and Hartman [19] study, an absence of re-evaluation immediately post-treatment prevented valid conclusions being drawn in relation to the effectiveness of the applied intervention. There are three potential underlying reasons for the decline in performance observed four months after termination of treatment in this study. One explanation is that any benefits of dysarthria therapy may have diminished over time, with peak effects occurring immediately after treatment [59]. Alternatively, speech exercise may have caused an exacerbation of dysarthria [16,17], or a third reason is that therapeutic gains may have been overshadowed by disease progression, whereby targeting a specific subsystem may have been of little or temporary benefit [60]. Without a comparative control group, a baseline observation period to characterise disease trajectory [22], and an evaluation immediately following intervention, definitive conclusions regarding the cause of speech decline cannot be reliably drawn.

In contrast, two contemporary studies presented preliminary findings that suggested possible benefits following integrated systems approaches, where all five speech subsystems were targeted during intervention simultaneously [45,46]. Significant methodological weaknesses within these studies, however, limited meaningful conclusions regarding intervention efficacy. The conceptual basis for these integrated approaches aligns with established theoretical perspectives. For example, Rosenbek and Jones [61] advocated for an integrated speech subsystems approach in cases where addressing the underlying pathophysiology proves unfeasible or futile. In ALS, the progressive nature of the disease encompasses an anticipated and inevitable deterioration across multiple speech subsystems, ultimately affecting respiratory support, laryngeal function, velopharyngeal control, and articulatory precision, and prosody. The rapid progression characteristic of this disease may have led researchers to conclude that addressing the underlying pathophysiology is futile, thereby necessitating a comprehensive intervention approach that simultaneously targets all affected speech components. Additionally, the motor speech treatment hierarchy recommends prioritising subsystems that support overall speech function, through hierarchically dependent, interactive stages where muscle tone, respiration, and phonation serve as foundational targets that directly influence the successful attainment of higher order targets of articulation and prosody [62]. These theoretical frameworks, whilst intuitive, require rigorous investigation through well-designed studies that can distinguish between intervention effects and natural disease progression, before they can inform clinical practice.

While frequency and dosage of intervention were specified by Rohers [46], the authors failed to adequately describe the treatment protocol relating to how the five integrated subsystems were targeted. Within the context of mixed dysarthria, this is especially important because approaches targeting flaccidity may potentially exacerbate presenting spasticity [15]. In addition, the study’s reliance on subjective patient-reported outcomes without clinician-ratings or objective acoustic measures of speech change represented a further methodological weakness. Although the reported improvements following speech treatment were encouraging in this case, without impairment or activity-based (e.g., intelligibility) speech measures in this study, the positive outcomes related to quality of life reported in this study could be attributed to non-specific effects such as therapeutic alliance or enhanced self-efficacy [63,64]. The authors’ interpretation of post-treatment FFR amplitude changes as indicating strengthened neural encoding potentially represents an overextension of the data, particularly as the neurophysiological mechanisms underlying such adaptations remain poorly understood [65]. Given these substantial limitations within a single-case design lacking appropriate controls, clinicians should not view this report as providing credible evidence supporting this intervention approach for patients with ALS.

In the second study utilising an integrated systems approach, Apreleva et al. [45] reported mean trends towards improvement or maintenance of speech function following a six week dysarthria treatment program. Despite these findings, methodological flaws within the study compromised the validity of the reported observations. Most critically, the exclusive use of descriptive statistics without inferential analysis combined with a small sample size, precluded the ability to determine whether observed changes were representative of intervention effects or alternatively, reflected natural performance or test-retest variability. A further limitation of this study was an absence of an activity-level speech outcome measure, such as an assessment of intelligibility. Although participant-reported speech subscores on the Centre for Neurologic Bulbar Function Scale (CNS-BFS) [66] offered insight into perceived speech function, they do not constitute a valid proxy for objective intelligibility assessments, which are fundamental to evaluating the outcome of dysarthria intervention [67].

### 4.2. Speech Treatment Hierarchy for Mixed Spastic-Flaccid Dysarthria

The complexity inherent in mixed dysarthria presentations in ALS, coupled with the limited empirical evidence supporting dysarthria intervention strategies, presents significant clinical challenges in identifying and implementing appropriate therapeutic targets for this population. To illustrate, the application of LSVT-LOUD^®^ in the Watts and Vanryckeghem [20] case, which demands high-effort, intensive exercise targeting vocal loudness, was contraindicated given co-occurring vocal hypo and hyperfunction [68]. The application of both LSVT-LOUD^®^ and isometric vocal exercises to address reduced volume and breathiness resulted in severe ventricular phonation. This presentation illustrates the clinical paradox that often arises in mixed dysarthria, whereby the treatment of one set of symptoms may inadvertently exacerbate others [15].

When mixed spastic and flaccid features are present in ALS, it may be appropriate to prioritise the management of spasticity first, as it can impose more immediate functional limitations on speech production. Indeed, Dworkin [69] posited a hierarchical treatment protocol for spastic dysarthria, recognising that premature force physiology training prior to tone normalisation may compound rather than remediate associated impairments. Vocal strain may also emerge as a compensatory response to respiratory weakness [70], therefore, therapeutic approaches that specifically adapt to declining respiratory capacity may prove most effective for optimizing voice production in ALS [71]. Indeed, this approach is supported by the motor speech treatment hierarchy which prioritises first-order targets of muscle tone and respiratory support as foundational elements underpinning effective second-order phonatory targets [62].

Speech treatment target selection should also be influenced by considerations of task specificity and the scope of intervention. For example, contemporary evidence supports global speech strategies (e.g., clear speech or reduced speech rate) for motor speech disorders that target a single parameter but yield effects across multiple subsystems [26,27,72]. In clinical practice, such strategies are often introduced as compensatory—measures when intelligibility begins to decline in people with ALS [73,74]. These strategies are typically applied reactively and not targeted systematically or as a component of repeated practice protocols. The proactive and sustained use of these approaches remains largely unexplored in ALS speech treatment protocols and warrants further investigation.

In contrast to these global approaches, Dworkin and Hartman [19] utilised tongue strengthening exercises in an effort to improve articulatory proficiency in a single case with ALS. Because weakness is the underlying neuromuscular issue for both spastic and flaccid dysarthria, strengthening exercises may be mistaken as being beneficial. The use of oral motor strengthening is controversial, however, and one of the main concerns is related to whole-part transfer in motor learning suggesting that gains from isolated tongue strengthening exercises may not translate to improved speech performance [75,76]—an issue that also applies to the Dworkin and Hartman [19] study. Indeed, recent research reported no significant relationship between maximum tongue pressure and intelligibility across different types of dysarthria, including mixed presentations [77]. These findings collectively undermine the theoretical basis for using non-speech strengthening approaches to manage dysarthria in ALS.

Beyond target selection, the manner in which intervention is implemented also requires careful consideration in ALS populations. Apreleva et al. [45] incorporated fatigue management through rest periods between exercises in a 6 week integrated subsystems music-based speech therapy program, an approach that theoretically aligns with recommendations for ALS populations [78]. Without a control group, robust statistical analysis, or comparison to natural history data, however, it is not possible to draw credible conclusions regarding the effectiveness of this approach on speech outcomes, as observed changes may reflect natural disease variability. While this study represents one of the first attempts to evaluate such approaches on dysarthria progression in ALS [18,79], it should not be viewed as providing evidence of treatment efficacy.

### 4.3. Heterogeneity of Bulbar Deterioration in Treatment Selection

The reviewed studies highlighted the potential significance of disease onset type and speech phenotype variation as a determinant of treatment success, despite limited systematic investigation of these factors. Notwithstanding the nature of interventions applied, impairment and activity-based speech outcome measures administered at multiple time points during a 4.5 month period of therapy, indicated the progressive and unremitting decline of speech function over time in the Watts and Vanryckeghem [20] case. This trajectory underscores the importance of identifying specific bulbar phenotypes early in the disease course [80], to establish potential therapeutic windows prior to the point where speech deterioration may outpace treatment benefit. This case highlights the broader methodological challenge of accounting for disease heterogeneity in intervention studies.

The Apreleva et al. [45] study detailed the exclusive inclusion of participants with early-to mid-stage disease, determined by ALSFRS-R bulbar subscores [47]. This stage-specific approach to recruitment reflects that intervention windows narrow as bulbar function progresses [81], though it should be noted that individuals within the same disease stage may present with markedly different patterns of bulbar involvement [4], necessitating individualised treatment approaches, rather than uniform intervention protocols. The delineation of disease stage is crucial to determine therapeutic windows based on dysarthria progression trajectories [82,83], enable phenotype-specific treatments [80], and establish realistic treatment expectations for both patient and clinician. Furthermore, defining appropriate windows for intervention will be crucial in shaping evidence-based guidelines around when to commence or discontinue active speech exercise in ALS, in favour of AAC approaches [84]. Ball et al. [83] recommend implementing AAC before speaking rate declines below 120 words per minute, after which a precipitous decline in speech function can be expected in individuals with ALS.

Despite the inclusion of disease stage in the Apreleva et al. [45] study, there were significant gaps in phenotype characterisation, which limited the interpretability of the findings. For example, the study failed to report the type and severity of dysarthria observed in the ALS participants, as well as primary subsystem impairments at baseline. Given that mixed dysarthria is commonly observed in ALS, it may be assumed that the 7 participants engaged in this study presented with mixed dysarthria. It is important to acknowledge that overall dysarthria severity and baseline speech characteristics may have been pivotal factors influencing the alterations in performance observed following this integrated systems intervention. An additional methodological weakness was a lack of reporting around participant attrition details. Originally, 8 participants were recruited into the study, comprised of 7 individuals with spinal onset disease and 1 individual with bulbar onset disease. One participant withdrew from the study and data was subsequently analysed and reported for 7 participants. The authors, however, neglected to specify disease onset type for the participant that withdrew from the study. This omission is significant as speech intervention efficacy in ALS may be linked to disease onset type, with spinal onset patients possibly deriving greater benefit due to greater bulbar integrity at the commencement of intervention [85], as well as a characteristically slower progression of bulbar symptoms [86]. Any observed maintenance of function in this study, therefore, may have simply reflected the natural history of a particular subgroup of ALS speakers rather than treatment effects.

In addition, Apreleva et al.’s [45] possible composition bias toward spinal-onset cases limits generalizability and potentially creates a misleading impression of treatment response. The potential exclusion of treatment-resistant presentations combined with the absence of statistical analysis should caution clinicians against viewing this study as providing evidence for this intervention approach, particularly for those with bulbar-onset disease or more advanced bulbar involvement. The 3 other studies evaluated in this review failed to specify disease-onset type. Symptom descriptions strongly suggest bulbar-onset disease in the Dworkin & Hartman [19] and Watts & Vanryckeghem [20] cases, however, the Roher’s et al. [46] study provided insufficient clinical detail to allow inferences about disease onset type in their participant with ALS. It is possible that this participant may have presented with spinal onset disease and taken together with the findings of the other studies in this review, may suggest that therapeutic potential for individuals with bulbar-onset disease is limited.

The current evidence base does not demonstrate that speech treatment is either effective or ineffective in ALS, but rather, it reveals a critical absence of sufficiently rigorous studies to draw any definitive conclusions about treatment outcomes. This equipoise creates both challenges and opportunities for clinical practice and research advancement. The theoretical underpinnings for early intervention show promise, with contemporary perspectives advocating for proactive intervention strategies that prioritise preservation and optimization of remaining bulbar function rather than delayed approaches that commence after significant functional decline [87]. This approach represents a shift from damage limitation to capacity building within a framework that acknowledges the progressive yet variable nature of the disease. Meta-analytic evidence examining post-exercise outcomes in ALS populations provides preliminary support for carefully designed therapeutic protocols that incorporate principles of motor learning and neuroplasticity, suggesting that appropriately structured interventions may yield measurable benefits without accelerating disease progression [87,88]. Translating these theoretical frameworks into clinically meaningful interventions requires addressing the fundamental methodological limitations that have plagued this field for decades. The sparse literature, marked by small samples sizes, heterogenous outcome measures, and absence of appropriate control conditions, underscores the critical need for systematic research that can distinguish between genuine treatment effects and natural disease variability.

## 5. Conclusions

This scoping review revealed a persistent and substantial gap in the evidence base for behavioural interventions targeting dysarthria in ALS, consistent with earlier reviews conducted over a decade ago. Only four studies were identified over a 46-year period, representing a critical gap in the research literature despite the prevalence and devastating impact of dysarthria in this population. The limited body of available research presented significant methodological weaknesses including insufficient reporting of clinical characteristics and intervention procedures, lack of appropriate statistical analysis, small sample sizes, an absence of comparative control groups, and variability in outcome measures employed. Despite claims of positive outcome trends following treatment in two contemporary studies, substantial methodological limitations hindered the ability to draw reliable conclusions regarding intervention effectiveness.

The theoretical rationale for comprehensive speech interventions that incorporate relaxation and fatigue management, including interspersed rest periods between exercise segments, rather than intensive strengthening in ALS has face validity, however, requires empirical validation. Whilst there is the suggestion that disease onset type and timing of intervention may influence outcomes, the current strength of evidence is not sufficient to support definitive clinical recommendations.

### 5.1. Limitations

This review had several limitations that should be considered when interpreting the findings. The search was restricted to six electronic databases and English language publications, potentially excluding relevant studies published in other languages and grey literature sources such as conference proceedings, theses, and unpublished reports. The exclusion of conference abstracts whilst justified by limited methodological detail, may have resulted in missing recent or ongoing research in this emerging field. Beyond these search-related constraints, the characteristics of the identified studies presented analytical constraints. The heterogeneity in study designs, outcome measures, and intervention approaches applied across the included studies largely precluded meaningful synthesis or meta-analysis of the findings.

### 5.2. Directions for Future Research

The above limitations, combined with the sparse evidence base and methodological weaknesses identified within the reviewed studies, highlight critical areas that must be addressed to advance the field of dysarthria management in ALS, and provide clinicians with reliable evidence to guide practice. Future treatment research must address several priorities, including: (1) adherence to established intervention reporting guidelines to ensure complete and transparent description of procedures, including materials, dosage, tailoring methods, and fidelity checks); (2) comprehensive documentation of participant characteristics (e.g., dysarthria type, severity, primary subsystem involvement, disease stage, and onset type) to establish which patients would benefit most from specific interventions and identify optimal timing for treatment; and (3) the standardisation of outcome measures across studies to enable meaningful comparison of treatment effects.

Methodologically, future studies should include extended baseline periods to better characterize individual disease trajectories prior to intervention, incorporate control or comparison conditions where feasible, and apply appropriate statistical methods suited to small sample sizes. Further investigation into phenotype-specific treatment responses is also needed, as is exploration of the proposed therapeutic window during which speech intervention may be optimally effective. Research should examine the long-term impact of early intervention on communication outcomes and identify optimal timepoints for transitioning from direct speech therapy to augmentative and alternative communication strategies.

Advancing this research agenda will be critical in shifting dysarthria care in ALS from reactive to proactive, empowering clinicians to intervene early, tailor treatment, and preserve natural speech and communication for as long as possible, in the face of progressive decline.

## Figures and Tables

**Table 1:** Studies investigating dysarthria treatment for motor neuron disease.

Author, Publication Date/ Country of Origin	Study Aims	Participants/ Sample Size	Study Design	Dysarthria Treatment	Speech Outcome Measure(s)	Measurement Timepoints	Key Findings	Level of Evidence (NHMRC)	JBI Critical Appraisal Criteria Met
Apreleva et al., 2022 (Russia) [45]	To test the feasibility and efficacy of a music therapy protocol designed to support bulbar and respiratory functions of individuals with early- and mid-stage ALS.	*N* = 7 (6 female, 2 male participants with ALS, mean age 58.1 yrs)	ABA mixed-methods case series with repeated measures	Individualised music therapy, 2x/week for 1 h, in-home, over 6 consecutive weeks. ALS-specific vocal health guidelines to promote healthy voice use/habits in daily life.	CNS-BFS (speech subscore), MPT (sec), AMR (syllables uttered), SMR (syllables uttered), Jitter (%), Shimmer (%), HNR (dB), VSA (Hz^2^), F0 (Hz), Speaking rate (words/min), Speech-pause ratio (oral reading; secs/min), Pause frequency (oral reading; pauses/min), Hypernasality level (perceptual rating). Thematic analysis of semi-structured interviews with participants and carers regarding treatment experience.	Baseline (week 1), pre-treatment (week 6), post-treatment (week 12), and after wash-out period (week 16).	Most bulbar & respiratory functions maintained or improved during treatment phase compared with control period (weeks 1 to 6)	III-3	70%
Dworkin & Hartman, 1979 (USA) [19]	To describe the effects of progressive lingual and velopharyngeal involvement on speech and swallowing over a 6-month period post-diagnosis, and outline the medical, speech, and prosthetic measures taken to lessen the effects of ALS.	*N* = 1 (Male with ALS, 49 yrs, mixed spastic-flaccid dysarthria)	Case report	Tongue strengthening program (resistance exercises) with regular articulation training; discontinued after one month due to fatigue/breathing difficulties.	Tongue strength; Lingual AMR; Perceptual ratings of articulatory precision, hypernasality, voice quality, intelligibility.	Baseline, 6 months post-treatment	Reductions in tongue strength and lingual AMR; Moderately imprecise articulation, hypernasality and nasal snorting progressed to severely affected; Mildly strain-strangled voice progressed to severely strain-strangled; Moderately affected intelligibility became severely affected.	IV	75%
Rohers et al., 2022 (Brazil) [46]	To assess the effectiveness of the frequency-following response (FFR) to monitor progress of speech therapy for dysarthria in neurodegenerative disease.	*N* = 2 (1 male with PD, 71 yrs, hypokinetic dysarthria; 1 male with ALS, 58 yrs, mixed spastic-flaccid dysarthria)	Descriptive, longitudinal, and qualitative pilot study	25 motor speech treatment sessions, 45 min each, over 6 months: 5 sessions targeted respiration, 5 targeted resonance, 5 targeted respiration & resonance together, 5 targeted prosody, and 5 targeted phonation.	Self-assessment questionnaire (Living with dysarthria); Electrophysiological assessment (FFR).	Pre- and post-treatment	Significant improvements in Living with Dysarthria scores for both participants across all domains. FFR –decreased latencies & increased amplitudes for some waves (both participants); responses more noticeable for amplitude measure.	IV	38%
Watts & Vanryckeghem, 2001 (USA) [20]	To present a case report of a female with bulbar ALS, and describe the effects of voice/speech and swallowing therapy.	***N*** = 1 (Female with ALS, 72 yrs, primary involvement of bulbar nerves, flaccid dysarthria)	Case report	Combination of Lee Silverman Voice Treatment (LSVT) and voice focus/resonant therapy to reduce laryngeal tension during initial stage of LSVT; 4x/week for 1 h, discontinued after 2 weeks. Voice focus therapy transitioning to articulation focus therapy over time; 1x/week for 1.5 hrs, over 4.5 months. Daily articulation therapy (motor motility & strengthening exercises).	Perceptual ratings of voice quality; Acoustic variables (F0 (Hz), Jitter (%), Shimmer (%), NHR (dB), vF0 (Hz), vAm (dB)); Sentence intelligibility ratings.	Pre-, during (monthly), and post-treatment	LSVT resulted in decline in voice quality (increased ventricular compression). No measurable/perceptible improvement in voice quality following voice focus/resonant therapy and resumption of LSVT. F0 increased during voice/articulation therapy while all other acoustic variables decreased over first 3 months/increased in last month of therapy. Intelligibility declined rapidly over course of therapy.	IV	88%

Key: ALS—Amyotrophic Lateral Sclerosis; CNS-BFS—Center for Neurologic Study Bulbar Function Scale; ALS—Amyotrophic Lateral Sclerosis; MPT—Maximum Phonation Time (seconds); AMR—Maximum repetition rate (Alternating); SMR—Maximum repetition rate (Sequential); HNR—Harmonics to Noise Ratio (Decibels); VSA—Vowel Space Area (Hertz squared); F0 (Hz)—Fundamental Frequency Hertz); FFR—Frequency-following response; PD—Parkinson’s disease; QOL—Quality of life; LSVT—Lee Silverman Voice Treatment; NHR—Noise to Harmonics Ratio (dB); vF0—F0 variation; vAm—peak amplitude variation; JBI = Joanna Briggs Institute.

## Data Availability

No new data were created or analyzed in this study. Data sharing is not applicable to this article.

## Supplementary Materials

The following supporting information can be downloaded at: https://www.mdpi.com/article/10.3390/healthcare13192434/s1, Supplementary File S1 (Preferred Reporting Items for Systematic reviews and Meta-Analyses extension for Scoping Reviews (PRISMA-ScR) Checklist, Supplementary File S2 (Final search strings).

## Abbreviations

The following abbreviations are used in this manuscript:

ALS	Amyotrophic Lateral Sclerosis
LSVT	Lee Silverman Voice Treatment
AAC	Augmentative and Alternative Communication
PRISMA	Preferred Reporting Items for Systematic Reviews and Meta-analyses
NHMRC	National Health and Medical Research Council
JBI	Joanna Briggs Institute
MT	Music Therapy
ALSFRS-R	ALS Functional Rating Scale- Revised
FFR	Frequency Following Response
ICF	International Classification of Functioning, Disability and Health
