# Supplementary material for: To Treat or Not to Treat: A Scoping Review of Speech Treatment for Dysarthria in Amyotrophic Lateral Sclerosis (ALS)
Source: Healthcare (Basel). 2025 Sep 25;13(19):2434. doi: 10.3390/healthcare13192434 (PMC12523638; doi:10.3390/healthcare13192434)
Supplement: Supplementary file 1 [file healthcare-13-02434-s001.zip › Supplementary File S2_Search Strings-tracked.pdf]

Supplementary File S2: MND/ALS & Dysarthria treatment - Final Search: 21/05/2025

PubMed: 1299 Results
("Motor Neuron Disease"[Mesh] OR "Amyotrophic Lateral Sclerosis"[Mesh] OR "motor neuron disease*"[tiab] OR "motor neurone disease*"[tiab] OR MND[tiab] OR "amyotrophic lateral sclerosis"[tiab] OR ALS[tiab] OR "lou gehrig*"[tiab] OR “progressive muscular atrophy”[tiab] OR “progressive bulbar palsy”[tiab] OR “lateral sclerosis*”[tiab] OR “neuromuscular disease*”[tiab])
AND
("Dysarthria"[Mesh] OR “Speech”[MeSH] OR “Articulation Disorders”[Mesh] OR “Communication Disorders”[Mesh] OR “Speech Disorders”[Mesh] OR “Voice Disorders”[Mesh] OR “Dysphonia”[Mesh] OR dysarthria*[tiab] OR “oculopharyngeal muscle dystrophy”[tiab] OR speech*[tiab] OR "speech disorder*"[tiab] OR "bulbar pals*"[tiab] OR "pseudobulbar pals*"[tiab] OR “laryngeal dysfunction*”[tiab] OR “hypernasality”[tiab] OR “articulation disorder*”[tiab] OR “communication disorder*”[tiab] OR “bulbar dysfunction”[tiab] OR dysarth*[tiab] OR dysphon*[tiab] OR anarth*[tiab] OR dyspros*[tiab] OR aphon*[tiab] OR dysfluent*[tiab])
AND
(“Language Therapy*”[Mesh] OR “Speech Therapy*”[Mesh] "Rehabilitation of Speech and Language Disorders"[Mesh] OR “speech therapy”[tiab] OR “speech pathology*”[tiab] OR therap*[tiab] OR intervention*[tiab] OR treatment*[tiab] OR rehabilitat*[tiab] OR management*[tiab] OR exercise*[tiab] OR clinic*[tiab] OR behavioural*[tiab] OR behavioral*[tiab])

CINAHL: 513 Results [used polygot translation tool]
((MH "Motor Neuron Disease+") OR (MH "Amyotrophic Lateral Sclerosis+") OR (TI "motor neuron disease*" OR AB "motor neuron disease*") OR (TI "motor neurone disease*" OR AB "motor neurone disease*") OR (TI MND OR AB MND) OR (TI "amyotrophic lateral sclerosis" OR AB "amyotrophic lateral sclerosis") OR (TI ALS OR AB ALS) OR (TI "lou gehrig*" OR AB "lou gehrig*") OR (TI "progressive muscular atrophy" OR AB "progressive muscular atrophy") OR (TI "progressive bulbar palsy" OR AB "progressive bulbar palsy") OR (TI "lateral sclerosis*" OR AB "lateral sclerosis*") OR (TI "neuromuscular disease*" OR AB "neuromuscular disease*"))
AND
((MH Dysarthria+) OR (MH Speech+) OR (MH "Articulation Disorders+") OR (MH "Communication Disorders+") OR (MH "Speech Disorders+") OR (MH "Voice Disorders+") OR (MH Dysphonia+) OR (TI dysarthria* OR AB dysarthria*) OR (TI "oculopharyngeal muscle dystrophy" OR AB "oculopharyngeal muscle dystrophy") OR (TI speech* OR AB speech*) OR (TI "speech disorder*" OR AB "speech disorder*") OR (TI "bulbar pals*" OR AB "bulbar pals*") OR (TI "pseudobulbar pals*" OR AB "pseudobulbar pals*") OR (TI "laryngeal dysfunction*" OR AB "laryngeal dysfunction*") OR (TI hypernasality OR AB hypernasality) OR (TI "articulation disorder*" OR AB "articulation disorder*") OR (TI "communication disorder*" OR AB "communication disorder*") OR (TI "bulbar dysfunction" OR AB "bulbar dysfunction") OR (TI dysarth* OR AB dysarth*) OR (TI dysphon* OR AB dysphon*) OR (TI anarth* OR AB a narth*) OR (TI dyspros* OR AB dyspros*) OR (TI aphon* OR AB aphon*) OR (TI dysfluent* OR AB dysfluen t*))
AND
((MH "Language Therapy*+") OR (MH "Speech Therapy*+") AND (MH "Language Disorders"+") OR (TI "speech therapy" OR AB "speech therapy") OR (TI "speech pathology*" OR AB "speech pathology*") OR (TI therap* OR AB therap*) OR (TI intervention* OR AB intervention*) OR (TI treatment* OR AB treatment*) OR (TI rehabilitat* OR AB rehabilitat*) OR (TI management* OR AB management*) O R (TI exercise OR AB exercise) OR (TI clinic* OR AB clinic*) OR (TI behavioural* OR AB behavioural*) OR (TI behavioral* OR AB behavioral*))

Embase: 3794 Results [used polygot translation tool]

#1- ('Motor Neuron Disease'/exp OR 'Amyotrophic Lateral Sclerosis'/exp OR 'motor neuron disease*':ti,ab OR 'motor neurone disease*':ti,ab OR MND:ti,ab OR 'amyotrophic lateral sclerosis':ti,ab OR ALS:ti,ab OR 'lou gehrig*':ti,ab OR 'progressive muscular atrophy':ti,ab OR 'progressive bulbar palsy':ti,ab OR 'lateral sclerosis*':ti,ab OR 'neuromuscular disease*':ti,ab)

#2- (Dysarthria/exp OR Speech/exp OR 'Articulation Disorders'/exp OR 'Communication Disorders'/exp OR 'Speech Disorders'/exp OR 'Voice Disorders'/exp OR Dysphonia/exp OR dysarthria*:ti,ab OR 'oculopharyngeal muscle dystrophy':ti,ab OR speech*:ti,ab OR 'speech disorder*':ti,ab OR 'bulbar pals*':ti,ab OR 'pseudobulbar pals*':ti,ab OR 'laryngeal dysfunction*':ti,ab OR hypernasality:ti,ab OR 'articulation disorder*':ti,ab OR 'communication disorder*':ti,ab OR 'bulbar dysfunction':ti,ab OR dysarth*:ti,ab OR dysphon*:ti,ab OR anarth*:ti,ab OR dyspros*:ti,ab OR aphon*:ti,ab OR dysfluent*:ti,ab)

#3- ('Language Therapy*'/exp OR 'Speech Therapy*'/exp AND 'Language Disorders'/exp OR 'speech therapy':ti,ab OR 'speech pathology*':ti,ab OR therap*:ti,ab OR intervention*:ti,ab OR treatment*:ti,ab OR rehabilitat*:ti,ab OR management*:ti,ab OR exercise:ti,ab OR clinic*:ti,ab OR behavioural*:ti,ab OR behavioral*:ti,ab)

#4- #1 AND #2 AND #3

Cochrane: 98 Results [used polygot translation tool]

([mh "Motor Neuron Disease"] OR [mh "Amyotrophic Lateral Sclerosis"] OR ("motor neuron" NEXT disease*):ti,ab OR ("motor neurone" NEXT disease*):ti,ab OR MND:ti,ab OR "amyotrophic lateral sclerosis":ti,ab OR ALS:ti,ab OR ("lou" NEXT gehrig*):ti,ab OR "progressive muscular atrophy":ti,ab OR "progressive bulbar palsy":ti,ab OR ("lateral" NEXT sclerosis*):ti,ab OR ("neuromuscular" NEXT disease*):ti,ab)
AND
([mh Dysarthria] OR [mh Speech] OR [mh "Articulation Disorders"] OR [mh "Communication Disorders"] OR [mh "Speech Disorders"] OR [mh "Voice Disorders"] OR [mh Dysphonia] OR dysarthria*:ti,ab OR "oculopharyngeal muscle dystrophy":ti,ab OR speech*:ti,ab OR ("speech" NEXT disorder*):ti,ab OR ("bulbar" NEXT pals*):ti,ab OR ("pseudobulbar" NEXT pals*):ti,ab OR ("laryngeal" NEXT dysfunction*):ti,ab OR hypernasality:ti,ab OR ("articulation" NEXT disorder*):ti,ab OR ("communication" NEXT disorder*):ti,ab OR "bulbar dysfunction":ti,ab OR dysarth*:ti,ab OR dysphon*:ti,ab OR anarth*:ti,ab OR dyspros*:ti,ab OR aphon*:ti,ab OR dysfluent*:ti,ab)
AND
([mh ("Language" NEXT Therapy*)] OR [mh ("Speech" NEXT Therapy*)] AND [mh "Language Disorders"] OR "speech therapy":ti,ab OR ("speech" NEXT pathology*):ti,ab OR therap*:ti,ab OR intervention*:ti,ab OR treatment*:ti,ab OR rehabilitat*:ti,ab OR management*:ti,ab OR exercise:ti,ab OR clinic*:ti,ab OR behavioural*:ti,ab OR behavioral*:ti,ab)

Web of Science: Advanced: 1191 Results [used polygot translation tool]
#1- ALL="Language Therapy*" OR ALL="Speech Therapy*" OR ALL="Language Disorders" OR (TI="speech therapy" OR AB="speech therapy") OR (TI="speech pathology*" OR AB="speech pathology*") OR (TI=therap* OR AB=therap*) OR (TI=intervention* OR AB=intervention*) OR (TI=treatment* OR AB=treatment*) OR (TI=rehabilitat* OR AB=rehabilitat*) OR (TI=management* OR AB=management*) OR (TI=exercise OR AB=exercise) OR (TI=clinic* OR AB=clinic*) OR (TI=behavioural* OR AB=behavioural*) OR (TI=behavioral* OR AB=behavioral*)
#2- ALL=Dysarthria OR ALL=Speech OR ALL="Articulation Disorders" OR ALL="Communication Disorders" OR ALL="Speech Disorders" OR ALL="Voice Disorders" OR ALL=Dysphonia OR (TI=dysarthria* OR AB=dysarthria*) OR (TI="oculopharyngeal muscle dystrophy" OR AB="oculopharyngeal muscle dystrophy") OR (TI=speech* OR AB=speech*) OR (TI="speech disorder*" OR AB="speech disorder*") OR (TI="bulbar pals*" OR AB="bulbar pals*") OR (TI="pseudobulbar pals*" OR AB="pseudobulbar pals*") OR (TI="laryngeal dysfunction*" OR AB="laryngeal dysfunction*") OR (TI=hypernasality OR AB=hypernasality) OR (TI="articulation disorder*" OR AB="articulation disorder*") OR (TI="communication disorder*" OR AB="communication disorder*") OR (TI="bulbar dysfunction" OR AB="bulbar dysfunction") OR (TI=dysarth* OR AB=dysarth*) OR (TI=dysphon* OR AB=dysphon*) OR (TI=anarth* OR AB=anarth*) OR (TI=dyspros* OR AB=dyspros*) OR (TI=aphon* OR AB=aphon*) OR (TI=dysfluent* OR AB=dysfluent*)
#3- ALL="Motor Neuron Disease" OR ALL="Amyotrophic Lateral Sclerosis" OR (TI="motor neuron disease*" OR AB="motor neuron disease*") OR (TI="motor neurone disease*" OR (AB="motor neurone disease*") OR (TI=MND OR AB=MND) OR (TI="amyotrophic lateral sclerosis" OR AB="amyotrophic lateral sclerosis") OR (TI=ALS OR AB=ALS) OR (TI="lou gehrig*" OR AB="lou gehrig*") OR (TI="progressive muscular atrophy" OR AB="progressive muscular atrophy") OR (TI="progressive bulbar palsy" OR AB="progressive bulbar palsy") OR (TI="lateral sclerosis*" OR AB="lateral sclerosis*") OR (TI="neuromuscular disease*" OR AB="neuromuscular disease*"))

#4- #1 AND #2 AND #3

Psycinfo: 354 Results [used polygot translation tool]
((DE "Motor Neuron Disease+") OR (DE "Amyotrophic Lateral Sclerosis+") OR (TI "motor neuron disease*" OR AB "motor neuron disease*") OR (TI "motor neurone disease*" OR AB "motor neurone disease*") OR (TI MND OR AB MND) OR (TI "amyotrophic lateral sclerosis" OR AB "amyotrophic lateral sclerosis") OR (TI ALS OR AB ALS) OR (TI "lou gehrig*" OR AB "lou gehrig*") OR (TI "progressive muscular atrophy" OR AB "progressive muscular atrophy") OR (TI "progressive bulbar palsy" OR AB "progressive bulbar palsy") OR (TI "lateral sclerosis*" OR AB "lateral sclerosis*") OR (TI "neuromuscular disease*" OR AB "neuromuscular disease*"))
AND
((DE Dysarthria+) OR (DE Speech+) OR (DE "Articulation Disorders+") OR (DE "Communication Disorders+") OR (DE "Speech Disorders+") OR (DE "Voice Disorders+") OR (DE Dysphonia+) OR (TI dysarthria* OR AB dysarthria*) OR (TI "oculopharyngeal muscle dystrophy" OR AB "oculopharyngeal muscle dystrophy") OR (TI speech* OR AB speech*) OR (TI "speech disorder*" OR AB "speech disorder*") OR (TI "bulbar pals*" OR AB "bulbar pals*") OR (TI "pseudobulbar pals*" OR AB "pseudobulbar pals*") OR (TI "laryngeal dysfunction*" OR AB "laryngeal dysfunction*") OR (TI hypernasality OR AB hypernasality) OR (TI "articulation disorder*" OR AB "articulation disorder*") OR (TI "communication disorder*" OR AB "communication disorder*") OR (TI "bulbar dysfunction" OR AB "bulbar dysfunction") OR (TI dysarth* OR AB dysarth*) OR (TI dysphon* OR AB dysphon*) OR (TI anarth* OR AB anarth*) OR (TI dyspros* OR AB dyspros*) OR (TI aphon* OR AB aphon*) OR (TI dysfluent* OR AB dysfluent*))
AND
((DE "Language Therapy*+") OR (DE "Speech Therapy*+") AND (DE "Language Disorders"+") OR (TI "speech therapy" OR AB "speech therapy") OR (TI "speech pathology*" OR AB "speech pathology*") OR (TI therap* OR AB therap*) OR (TI intervention* OR AB intervention*) OR (TI treatment* OR AB treatment*) OR (TI rehabilitat* OR AB rehabilitat*) OR (TI management* OR AB management*) OR (TI exercise OR AB exercise) OR (TI clinic* OR AB clinic*) OR (TI behavioural* OR AB behavioural*) OR (TI behavioral* OR AB behavioral*))
